# Novel *Klebsiella pneumoniae* K23-Specific Bacteriophages From Different Families: Similarity of Depolymerases and Their Therapeutic Potential

**DOI:** 10.3389/fmicb.2021.669618

**Published:** 2021-08-09

**Authors:** Roman B. Gorodnichev, Nikolay V. Volozhantsev, Valentina M. Krasilnikova, Ivan N. Bodoev, Maria A. Kornienko, Nikita S. Kuptsov, Anastasia V. Popova, Galina I. Makarenko, Alexander I. Manolov, Pavel V. Slukin, Dmitry A. Bespiatykh, Vladimir V. Verevkin, Egor A. Denisenko, Eugene E. Kulikov, Vladimir A. Veselovsky, Maja V. Malakhova, Ivan A. Dyatlov, Elena N. Ilina, Egor A. Shitikov

**Affiliations:** ^1^Department of Molecular Biology and Genetics, Federal Research and Clinical Center of Physical-Chemical Medicine, Moscow, Russia; ^2^Department of Molecular Microbiology, State Research Center for Applied Microbiology and Biotechnology, Moscow, Russia; ^3^Research Center of Biotechnology of the Russian Academy of Sciences, Winogradsky Institute of Microbiology, Moscow, Russia

**Keywords:** *Klebsiella pneumoniae*, multidrug-resistance, capsular type, capsule depolymerase, bacteriophage, *Galleria mellonella*

## Abstract

Antibiotic resistance is a major public health concern in many countries worldwide. The rapid spread of multidrug-resistant (MDR) bacteria is the main driving force for the development of novel non-antibiotic antimicrobials as a therapeutic alternative. Here, we isolated and characterized three virulent bacteriophages that specifically infect and lyse MDR *Klebsiella pneumoniae* with K23 capsule type. The phages belonged to the *Autographiviridae* (vB_KpnP_Dlv622) and *Myoviridae* (vB_KpnM_Seu621, KpS8) families and contained highly similar receptor-binding proteins (RBPs) with polysaccharide depolymerase enzymatic activity. Based on phylogenetic analysis, a similar pattern was also noted for five other groups of depolymerases, specific against capsule types K1, K30/K69, K57, K63, and KN2. The resulting recombinant depolymerases Dep622 (phage vB_KpnP_Dlv622) and DepS8 (phage KpS8) demonstrated narrow specificity against *K. pneumoniae* with capsule type K23 and were able to protect *Galleria mellonella* larvae in a model infection with a *K. pneumoniae* multidrug-resistant strain. These findings expand our knowledge of the diversity of phage depolymerases and provide further evidence that bacteriophages and phage polysaccharide depolymerases represent a promising tool for antimicrobial therapy.

## Introduction

*Klebsiella pneumoniae* is a widespread Gram-negative, non-motile, facultative anaerobic bacterium naturally occurring in soil, sewage, or plants. These bacteria are traditionally considered as commensals and can be found on human skin and in the gastrointestinal and respiratory tracts (Paczosa and Mecsas, 2016). Despite this, *K. pneumoniae* is the world’s second most common nosocomial pathogen, capable of causing a wide range of infections such as septicemia, pneumonia, urinary tract infections, and surgical- and catheter-related infections (Podschun and Ullmann, 1998).

The extensive use of antibiotics to treat *Klebsiella*-associated infections has led to the emergence and spread of drug resistance. According to data from the European Centre for Disease Prevention and Control, nearly 40% of the *K. pneumoniae* isolates in Europe are resistant to at least one class of antibiotic – fluoroquinolones, third-generation cephalosporins, aminoglycosides, or carbapenems (European Centre for Disease Prevention and Control, 2019). Of these, the isolates carrying extended-spectrum beta-lactamases or carbapenemases encoding genes are considered especially difficult to treat. As previously reported, these isolates cause infections with high morbidity and mortality rates (Cantón et al., 2012; Tzouvelekis et al., 2012; Munoz-Price et al., 2013; Girmenia et al., 2015; Tängdén and Giske, 2015; Lee et al., 2016).

In light of the antibiotic resistance crisis, the development of new approaches to antimicrobial therapy is especially relevant. One promising alternative to the use of antibiotics is therapy with virulent bacteriophages (phages; Górski et al., 2020). Phages are natural killers that can rapidly and selectively infect and lyse pathogenic bacteria, including *K. pneumoniae* clones associated with antibiotic resistance. Bacteriophage therapy has been clinically used since the beginning of the 20th century, and no known significant side effects have been identified throughout the entire history of use (Payne and Jansen, 2000). Nowadays, the efficiency and safety of bacteriophage therapy have been confirmed in mammal and *Galleria mellonella* models and in humans (Chhibber et al., 2008; Hung et al., 2011; D’Andrea et al., 2017; Manohar et al., 2019). The natural limitation of the approach is the usually narrow host range of individual bacteriophages, so the phage cocktails have to be employed to combat an unknown pathogen (Clark and March, 2006).

The host range of *K. pneumoniae* phages shows a good correlation with the type of capsular polysaccharide (CPS; Pires et al., 2016). To date, at least 130 types of CPS have been described, which is the key virulence factor protecting the bacteria from the immune system and the action of some antibiotics (Stewart, 1996; Yu et al., 2015; Wyres et al., 2016). Isolates characterized by the overexpression of capsular polysaccharides often possess an enhanced virulence and have been categorized as a separate group called hypervirulent *K. pneumoniae* (hvKp). In addition to hyperexpression of the capsule, frequently regulated by the rmpA/rmpA2 gene, hvKp may also carry other virulence factors, such as yersiniabactin, colibactin, aerobactin, and salmochelin. HvKp has been described as a cause of primary purulent liver abscesses in the Asia-Pacific region (Liu et al., 1986; Fang et al., 2005; Turton et al., 2007; Shon et al., 2013).

The main factor that allows phages to infect the *K. pneumoniae* cell is the presence of a specific receptor-binding protein (RBP) – a processive enzyme with depolymerase activity (Pires et al., 2016). Depolymerases are enzymes that can cleave capsular polysaccharides, clearing the way for phage adsorption to the surface of a bacterial cell, followed by injection of phage DNA. Phage-borne depolymerases are usually presented as a structural component of the phage adsorption apparatus (tail fibers, tail spikes, or base plates; Pires et al., 2016; Knecht et al., 2020). Most of the phage genomes encode only one depolymerase, but some phages have two or more depolymerases that allow them to infect multiple capsular types (Pan et al., 2017; Latka et al., 2019). Characterizing the diversity of phage depolymerases is of particular interest, as this could lead to the development of a new class of antivirulence agents. It has been shown that phage depolymerases could increase the rate of bacterial killing by serum *in vitro* and significantly increase survival in mice and *G. mellonella* larvae models (Majkowska-Skrobek et al., 2018; Solovieva et al., 2018; Volozhantsev et al., 2020). Additionally, phage-borne depolymerases can be used for the rapid determination of microbial capsule types or disruption of bacterial biofilms (Scorpio et al., 2008).

Currently, over 30 specific polysaccharide-depolymerases have been characterized (Lin et al., 2014; Majkowska-Skrobek et al., 2016, 2018; Hsieh et al., 2017; Pan et al., 2017, 2019; Latka et al., 2019; Domingo-Calap et al., 2020a,b; Liu et al., 2020; Volozhantsev et al., 2020). However, phages and depolymerases specific for *K. pneumoniae* K23 capsular type have not been characterized yet. In this study, we described the biological characteristics and performed a genomic analysis of one new podovirus vB_KpnP_Dlv622 and two new myoviruses (vB_KpnM_Seu621 and KpS8) infecting specifically the strains of *K. pneumoniae* with capsule type K23. In addition, we compared their depolymerases and assayed their protective activity in *G. mellonella* larvae during infection with a clinical multidrug-resistant (MDR) *K. pneumoniae* strain.

## Materials and Methods

### Bacterial Strains and Their Characterization

A collection comprising 32 clinical *K. pneumoniae* isolates from the Clinical Hospital №123 (Odintsovo, Russia) and 51 strains from the State Collection of Pathogenic Microorganisms and Cell Cultures, SCPM-Obolensk (State Research Center for Applied Microbiology and Biotechnology, Obolensk, Russia) were included in this study (Supplementary Table 3). All bacteria were grown in the Nutrient Medium No. 1 (SRCAMB, Obolensk, Russia), or in the lysogeny broth (LB) medium (Himedia, India) at 37°C. Bacterial identification was performed by MALDI-TOF mass spectrometry as described previously (Kornienko et al., 2016). The antibiotic susceptibility was tested using the disc diffusion method according to Clinical and Laboratory Standards Institute guidelines 28th edition (Clinical and Laboratory Standards Institute, 2018). Multilocus sequence typing (MLST) of *K. pneumoniae* strains was performed by determining the nucleotide sequences of seven housekeeping genes as described previously (Diancourt et al., 2005). The capsular type was determined by *wzi* gene sequencing (Brisse et al., 2013).

### Bacteriophage Isolation and Purification

Two *K. pneumoniae* K23 strains (Kp-9068 and KPi4275) were used as hosts for bacteriophage isolation. Phages vB_KpnM_Seu621 and vB_KpnP_Dlv622 were isolated from the Chermyanka river water samples and phage KpS8 was isolated from sewage by a previously described method (Van Twest and Kropinski, 2009), with slight modifications. In brief, 15 ml of sewage or river water samples were centrifuged at 10,000 *g* for 15 min, and the supernatant was filtered using a 0.22-μm sterile membrane syringe filter (Millipore, United States). Filtered supernatant and 0.2 ml of early log-phase host cultures (OD_600nm_ = 0.3) were added to 15 ml of double-concentrated LB broth and incubated overnight with agitation (200 rpm) at 37°C to amplify the phages. Further, the culture was centrifuged at 10,000 *g* for 15 min and subsequently filtered through 0.22-μm filters. Obtained lysates were serially diluted in LB and spotted on double-layer agar plates of host-strains for phage detection and isolation. Three rounds of single plaque purification and re-infection of exponentially growing host strains yielded pure bacteriophage suspensions. Bacteriophage titers were determined using a double-agar overlay plaque assay (Mazzocco et al., 2009).

### Electron Microscopy of Phage Particles

Purified phage preparations were analyzed by transmission electron microscopy using a JOEL JSM 100 CXII electron microscope (JOEL, Japan) at an acceleration voltage of 100 kV with a Gatan Erlangshem CCD camera (Gatan, Inc.). Carbon-coated grids with collodion supporting film were negatively stained with 1% uranyl acetate in methanol.

### Host Range Determination

The host range of phages was determined by the spot test using 83 *K. pneumoniae* strains. Briefly, 100 μl of log-phase (OD_600nm_ = 0.3) culture of each strain was added to 5 ml of top agar, which was subsequently poured onto a bottom agar plate. Around 5 μl of phage lysate at a titer of 10^6^ PFU/ml were spotted onto freshly seeded lawns of the strains and left to dry before overnight incubation at 37°C. Additionally, the efficiency of plating (EOP) assay was also performed for phage-sensitive strains as previously described (Solovieva et al., 2018). The average EOP value for a phage-bacterium ratio was classified according to Mirzaei and Nilsson (2015): highly productive (EOP ≥ 0.5), medium productive (0.1 ≤ EOP < 0.5), low productive (0.001 < EOP < 0.1), or inefficient (EOP ≤ 0.001; Horváth et al., 2020).

### Thermal Tolerance of Phages

The stability of phages at different temperatures was determined by incubating 1 ml of phage lysate (10^9^ PFU/ml) at different temperatures (4, 37, 45, 55, 65, and 75°C) for 1 h, and then the phage titer was determined using the double-layer agar method (Mazzocco et al., 2009). The assay was performed in triplicate.

### Adsorption Assay and One-Step Growth Curve

The adsorption rate was estimated as described earlier with a slight modification (Kropinski, 2009). Host strains were grown to exponential phase (OD_600nm_ = 0.3) and mixed with bacteriophage at a multiplicity of infection (MOI) of 0.01. Every 2 min from 2 to 17 min, aliquots were taken and treated with 2% chloroform, shaken briefly and set aside for 10 min at RT. The titers of free phage were quantified by plaque assay. Determining the number of PFU of the unbound phage in the supernatant and subtracting it from the total number of inputs PFU gave phage adsorption percentage.

The dynamic changes in the number of phage particles during a replicative cycle were performed as described earlier (Ciacci et al., 2018). Aliquots of phage lysates were added to an exponential phase host strains (OD_600nm_ = 0.3) in MOI of 0.01 and allowed to adsorb for 10 min at 37°C. The mixture was then centrifuged at 10,000 *g* for 5 min and the pellet was resuspended in 10 ml of LB broth to remove free phage particles. Aliquots were taken at 15, 20, 40, 50, 60, 70, and 80 min post-infection and treated with 2% chloroform, shaken briefly, set aside for 10 min at RT, and centrifuged. Finally, the titers of the lysates were quantified by plaque assay. The burst size was calculated as the ratio of the final count of released phage particles to the initial count of infected bacterial cells during the latent period. Independent experiments were repeated three times.

### DNA Sequencing and Analysis

A standard phenol-chloroform extraction protocol was used for phage DNA isolation as described previously (Green et al., 2012). The whole-genome sequencing of bacteriophages was performed with a high-throughput Illumina HiSeq system. The assembly was performed using SPAdes (v.3.14.0; Bankevich et al., 2012). Terminal repeats were predicted with the PhageTerm tool (Garneau et al., 2017). Open reading frames (ORFs) were predicted using GeneMarkS (version 4.32; Besemer et al., 2001), Phast (Zhou et al., 2011), and VGAS (Zhang et al., 2019). tRNAScan-SE (Chan and Lowe, 2019) and ARAGORN (Laslett and Canback, 2004) were used to predict tRNA. The putative functions of the proteins encoded by each ORF were retrieved manually using BLASTp, HHPred, Phast, and InterPro. ORFs were also compared against Virulence Factors of Pathogenic Bacteria (VFDB; Liu et al., 2019) and Antibiotic Resistance Genes Databases (ARDB; Liu and Pop, 2009) to verify the safety of the phages. The annotated genome sequences of bacteriophages vB_KpnM_Seu621, vB_KpnP_Dlv622, and KpS8 were deposited in the NCBI GenBank database under accession numbers MT939253, MT939252, and MT178275, respectively. Phylogenetic analysis was performed using the amino acid sequences of RNA polymerase for *Autographiviridae* family phages and major capsid protein for *Myoviridae* family phages as recommended by the International Committee on Taxonomy of Viruses (ICTV) classification. The phylogenetic tree was constructed with the Genome-BLAST Distance Phylogeny method implemented by the VICTOR webserver (Meier-Kolthoff and Göker, 2017). Depolymerase domain multiple sequences alignment was performed using MAFFT (v.7.475; Katoh and Standley, 2013). ProtTest (v.3.4.2) was used to define the best suitable amino acid substitution model (Darriba et al., 2011). Maximum likelihood trees were inferred with RAxML-NG (v.1.0.2) with Blosum62+G+F substitution model suggested by ProtTest (Kozlov et al., 2019). Trees were visualized with the ggtree package for R (Yu et al., 2017).

### Preparation of Recombinant CPS Depolymerases and Determination of Their Activity

To obtain recombinant CPS depolymerases, coding sequences for the genes kps8_053 and dlv622_00059 containing the putative depolymerase domains were amplified by PCR using specific oligonucleotide primer pairs: DepS8F_NcoI 5'-ACGCCATGGACTGGGTCACTCTTGAAAT-3' and DepS8R_XhoI 5'-ATACTCGAGCCCGTTCACCCTTGAAA-3'; Dep622F_NcoI 5'-TACCATGGCTTTGACAAAGTTAGTAC-3' and Dep622R_XhoI 5'-TACTCGAGCACCCCCGTCAACCGC-3'. Amplified fragments were cloned into a pET22b expression vector (Novagen, United States) *via* the *NcoI* and *XhoI* restriction sites and then transformed into *Escherichia coli* BL21 (DE3). Resulting constructs were quality-checked *via* Sanger sequencing. Protein expression was performed in LB medium supplemented with ampicillin at 100 mg/L. Transformed cells were grown at 37°C until the optical density reached the value of 0.4 at 600 nm. The medium was cooled to the temperature of 16°C followed by expression induction with 1 mM isopropyl-1-thio-β-D-galactopyranoside (IPTG). After further incubation at 16°C overnight, the cells were harvested by centrifugation at 3,700 *g* for 20 min, 4°C. The cell pellets were resuspended in 1/50 of the original cell volume in buffer A (20 mM Tris pH 8.0, 0.5 M NaCl, 20 mM imidazole) and then lysed by sonication. The cell debris was removed by centrifugation at 16,000 *g* for 30 min, at 4°C. The supernatants were loaded onto nickel Ni^2+^-charged 5 ml GE HisTrap columns (GE Healthcare Life Sciences) equilibrated with buffer A, and eluted with a 20–500 mM imidazole linear gradient in buffer A. The fractions containing the target proteins were pooled together and then dialyzed against 20 mM Tris pH 8.0, 200 mM NaCl, 0.5 mM DTT buffer at 4°C. The protein samples were concentrated with Sartorius ultrafiltration devices (molecular weight cutoff of 10,000) and stored at 4°C. The CPS-degrading activity of the recombinant proteins was assayed in а spot test using *K. pneumoniae* strains of different capsular types.

### Phage KpS8 and vB_KpnP_Dlv622 Adsorption Inhibition Assay

Bacterial cell suspension (~2 × 10^8^ CFU/ml) in SM-buffer (8 mM MgCl_2_, 100 mM NaCl, 50 mM Tris-HCl pH 7.5) with added recombinant depolymerase (400 μg/ml) and a phage sample (~10^7^ PFU/ml) were first brought to 37°C. Then equal volumes of the phage and cell suspension were mixed. After 5 min incubation at 37°C, bacterial cells with adsorbed phages were precipitated by centrifugation at 14,000 *g* for 1 min and the titer of non-adsorbed phages was determined in the supernatant.

### *Galleria mellonella* Larvae Infection Model

Greater Wax moth larvae (*G. mellonella*) were obtained from a laboratory culture maintained at State Research Center for Applied Microbiology and Biotechnology, Obolensk. The larvae were reared on an artificial nutrient medium (maize flour – nine parts, wheat flour – four parts, dry brewer’s yeasts, dry milk, beeswax, bee honey, and glycerol – five parts each) at 27°C for 25–27 days and were subsequently selected for the experiments. The larvae were inoculated with a bacterial suspension or bacteria simultaneously with the depolymerase enzyme into the hemocoel using an insulin syringe. Bacterial suspensions of an overnight agar *K. pneumoniae* Kp-9068 culture in phosphate-buffered saline (PBS) buffer with cell concentration of 3 × 10^8^ CFU per injection, 3 × 10^7^ CFU, 3 × 10^6^ CFU, 3 × 10^5^ CFU, and 3 × 10^4^ CFU were used to infect the larvae. Three larvae groups were used in the experiments: (1) larvae infected with bacteria only; (2) larvae infected with bacteria and DepS8 enzyme; and (3) larvae infected with bacteria and Dep622 enzyme. Each test was performed in triplicate, with 50 larvae per trial (10 larvae for each bacterial dose). The final dose of the enzymes was 2 μg per larvae for all trials. In addition, three control groups were used: larvae injected with PBS and larvae injected with depolymerase DepS8 or Dep622. Infected larvae were incubated at 37°C for 5 days and mortality was recorded daily. The GraphPad Prism software (GraphPad Software, Inc., La Jolla, United States) was used for statistical analysis and graphical presentation of the results. Statistical analysis was performed for pairwise comparisons between larvae infected with bacteria only and larvae infected with bacteria simultaneously with depolymerase DepS8 or Dep622 using log-rank (Mantel-Cox) test. Values of *p* < 0.05 were considered as statistically significant.

A dose of bacteria that is sufficient to kill 50% of a larvae population (LD_50_) was calculated from the cumulative mortality observed 5 days after dosing by the Ashmarin-Vorobiev modification of Karber’s method (Ashmarin and Vorobyev, 1962).

## Results

### Isolation and Phenotypic Characterization of Three Novel *Klebsiella pneumoniae* Phages

Phages vB_KpnM_Seu621 and vB_KpnP_Dlv622 were isolated in 2018 from the freshwater of the Chermyanka river (Moscow, Russia). Phage KpS8 was isolated in 2016 from sewage samples in the Moscow region, Russia. *Klebsiella pneumoniae* strain Kp-9068 was used as a host for isolation vB_KpnM_Seu621 and vB_KpnP_Dlv622 phages. The KpS8 phage was isolated using the KPi4275 strain as a host. Both strains had a multidrug-resistant phenotype (resistance to three or more classes of antibiotics), belonged to sequence type 11 (ST11), and had a K23 capsular type (Supplementary Table 3).

**Figure 1:**
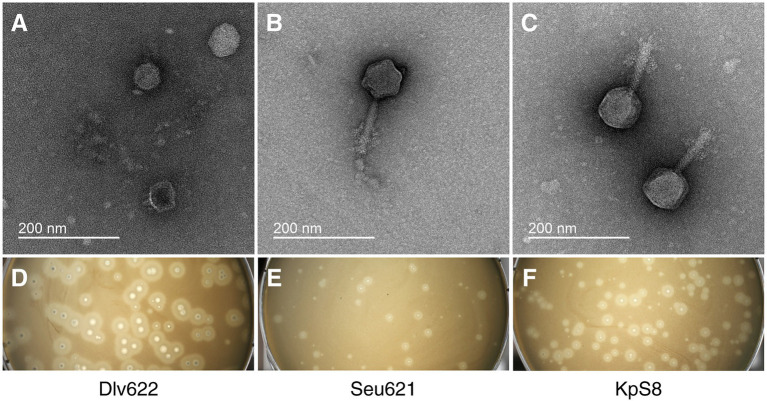
Initial characterization of *Klebsiella* phages vB_KpnP_Dlv622, vB_KpnM_Seu621, and KpS8. Transmission electron microscopy of phages Dlv622 **(A)**, Seu621 **(B)**, and KpS8 **(C)**. Plaque morphology of phages Dlv622 **(D)**, Seu621 **(E)**, and KpS8 **(F)**.

Phage vB_KpnP_Dlv622 had morphological features typical of the *Podoviridae* family: symmetrical polyhedral head (51 nm in diameter) and a short, noncontractile tail (12 nm long; Figure 1A). Both vB_KpnM_Seu621 and KpS8 phages, had an isometric head of 75–78 nm in diameter and a contractile tail of 104–113 nm in length, with the overgrowths on a terminal side (Figures 1B,C), suggesting that the phages belong to the *Myoviridae* family.

Different plaque morphology was exhibited by the phages. The vB_KpnP_Dlv622 phage formed clear circular plaques with a diameter of about 2–3 mm with halos (Figure 1D). Phages vB_KpnM_Seu621 and KpS8 were able to form similar circular plaques with a diameter of about 0.5–1 mm, surrounded by hazy halos (Figures 1E,F). Previous studies have suggested that the translucent halos were related to a putative phage-derived polysaccharide depolymerase (Wang et al., 2019).

Studies on phage adsorption have shown that all phages were able to adsorb to the Kp9068 strain by 6–7 min of incubation (Supplementary Figure 1A). The results of a one-step growth assay revealed similar latency and burst periods for all three phages of approximately 30 min (Supplementary Figure 1B). The burst sizes were approximately 66 PFU/cell for the vB_KpnP_Dlv622 phage and 85 and 96 PFU/cell for the vB_KpnM_Seu621 and KpS8, respectively.

The stability test of vB_KpnP_Dlv622, vB_KpnM_Seu621, and KpS8 phages showed that the phages were relatively stable in a range of temperature between 4 and 55°C (Supplementary Figure 2). vB_KpnM_Seu621 and KpS8 phages retained high plaque-forming activity even after incubation at 65°C, indicating good thermal stability. Incubation at a temperature of 75°C demonstrated a significant decrease in titer of all bacteriophages from 10^9^ PFU/ml to 10^2^–10^5^ PFU/ml.

### Genome Sequencing and Comparative Genomics

The complete genome of the vB_KpnP_Dlv622 phage was 44,687 bp long, with a G + C content of 54% and two 278 bp long direct repeats located at both ends. Bioinformatic analysis revealed 59 ORFs with a total length of 41,477 bp (coding percentage, 92.8%). BLAST analysis showed that this phage belongs to the *Autographiviridae* family. To verify this, amino acid sequences of RNA polymerase were used to estimate phylogenetic relationships between vB_KpnP_Dlv622 phage and 46 phages recommended by ICTV and belonging to the *Autographiviridae* family (last accessed April 23, 2020; Supplementary Figure 3). According to the phylogenetic tree, the vB_KpnP_Dlv622 phage belongs to the *Drulisvirus* genus of the family *Autographiviridae* and most closely related to the phage phiKpS2 (accession NC_047857.1, 82% query coverage and 92.6% identity according to BLASTn).

The genome of vB_KpnM_Seu621 was a linear dsDNA molecule of 142,896 bp with a G + C content of 44.64%. A total of 274 ORFs were identified. The complete genome of the KpS8 phage was also a linear dsDNA molecule of 143,800 bp (44.64% G + C content) containing 285 ORFs. Most of the predicted ORFs for both vB_KpnM_Seu621 and KpS8 phages were encoded on the positive strand. According to the Average Nucleotide Identity (ANI; Rodriguez-R and Konstantinidis, 2014) analysis, vB_KpnM_Seu621 and KpS8 phages had high ANI value of 99.48%. According to the BLAST analysis, phages belonged to the *Myoviridae* family. To verify the taxonomic position of the phages, the phylogenetic analysis was performed using multiple alignments of amino acid sequences of major capsid protein of vB_KpnM_Seu621 and KpS8 phages, 24 phages recommended by ICTV, and four the most related phages from the NCBI database. The results of the phylogenetic analysis showed that our phages are closely related to phage vB_KpnM_KB57 (accession NC_028659.1, 90% query coverage and 98.9% identity according to BLAST) and formed a distinct branch within the clade of *Mydovirus* genus, *Vequintavirinae* subfamily (Supplementary Figure 4).

### Functional Annotation of *K. pneumoniae* Phages

According to functional analysis, the proteins of the vB_KpnP_Dlv622 phage could be attributed to five groups: (1) phage structure (11 ORFs); (2) replication, regulation, transcription, and translation (20 ORFs); (3) DNA packaging (two ORFs); (4) host lysis (three ORFs); and (5) hypothetical protein (23 ORFs). vB_KpnP_Dlv622 had typical T7-related genome organization with the presence of lysis cassette composed of spanin-, holin-, and endolysin-encoding genes located next to each other, as well as phage DNA- and RNA-polymerases. No tRNA genes were identified with tRNAScan-SE and ARAGORN toolsets, as well as no significant similarities with known antibiotic resistance determinants, virulence or toxin proteins, and integrase genes were revealed (Supplementary Table 1A).

The genome of vB_KpnP_Dlv622 phage encoded two putative tail fiber and spike proteins dlv622_orf00051 and dlv622_orf00059. The dlv622_orf00051 was 317 aa long and was highly conserved among phages of the *Drulisvirus* genus. In contrast, dlv622_orf00059 showed more than 80% similarity only to a few phage proteins available in the NCBI database and encoded the potential pectate lyase domain with beta-helix structure characteristic of CPS depolymerases. According to a previous study, this structure of tail fiber and spike proteins means that phage vB_KpnM_Dlv622 belongs to Group A of KP34 viruses containing only one receptor-binding protein dlv622_orf00059 (Table 1; Latka et al., 2019).

**Table 1:** Polysaccharide depolymerase motifs identified in phage fiber and spike proteins.

Phage	Gene (Protein ID)	Protein size, aa	Motif, aa	Protein family	Analysis tool	Database identifier	E-value
vB_KpnP_Dlv622	Dlv622_orf00059 (QOI68577.1)	555	32–319	Pectin lyase fold	InterPro	IPR012334	3.87e-06
			24–445	Beta-helix, tailspike, Lyase	HHpred	1RMG_A	1.9e-16
vB_KpnM_Seu621 and KpS8	Seu621_orf00052 (QOI68629.1) and KpS8_053 (YP_009859099.1)	607	74–373	Beta-helix, tailspike, Lyase	HHpred	4RU5_B	4.3e-14
			80–364	Pectin lyase fold	InterPro	IPR012334	8.90e-05

For the vB_KpnM_Seu621 and KpS8 phages, a specific putative function (structural proteins, enzymes involved in the replication, regulation, transcription, and translation of DNA, host lysis) could be assigned to products of the same number of ORFs (*N* = 78). About 24 tRNA were detected in each bacteriophage by tRNAScan-SE and confirmed by ARAGORN. No genes related to phage lysogeny were predicted (Supplementary Tables 1B,C).

Based on the annotations of vB_KpnM_Seu621 and KpS8 phages, five ORFs are likely involved in tail fiber synthesis: (1) putative tail fiber protein (phage621_orf00040 and kps8_orf041), (2) putative tail fiber protein (phage621_orf00045 and kps8_orf046), (3) L-shaped tail fiber (phage621_orf00048 and kps8_orf049), (4) putative tail spike protein (phage621_orf00050 and kps8_orf051), and (5) putative tail spike protein (phage621_orf00052 and kps8_orf053). All the genes involved in tail fiber synthesis were either identical in both phages or had single amino-acid substitutions (phage621_orf00040 and kps8_orf041). It should be noted that among the ORFs described above phage621_orf00052/kps8_orf053 had a high score of similarity with dlv622_orf00059 (98% query coverage and 65.98% identity according to BLASTp; Supplementary Figure 5). In addition, it was revealed that these proteins possess the pectate lyase domain and beta-helix structure characteristic of CPS depolymerases (Table 1).

### Host Range of the Three *Klebsiella pneumoniae* Phages

The panel of 83 *K. pneumoniae* strains was used to determine the host ranges of the phages. Antimicrobial susceptibility profiles revealed that 53 strains had a multidrug-resistant phenotype (resistance to three or more classes of antibiotics), including at least 19 carbapenem-resistant strains. Results of multilocus sequence typing and *wzi* gene sequencing showed that *K. pneumoniae* strains belong to 21 different sequence types and had 26 unique capsular types, respectively (Supplementary Table 3). The most common CPS-types were K2 (*n* = 14; 16.5%), K57 (*n* = 11; 12.9%), KL39 (*n* = 9; 10.6%), and K1 (*n* = 8; 9.4%).

Isolated phages had a narrow range of lytic activity against *K. pneumoniae* strains (Supplementary Table 3) and could only infect and lyse strains belonging to the K23 (4/4) capsular types. According to EOP analysis, *Myoviridae* phages vB_KpnM_Seu621 and KpS8 revealed a high productive infection for three of the four K23 strains, whereas phage vB_KpnP_Dlv622 showed similar effectiveness only on its original host strain (Table 2).

**Table 2:** The efficiency of plating and depolymerase activity for phages KpS8, vB_KpnM_Seu621, and vB_KpnP_Dlv622 phages and their recombinant depolymerases.

Strain	*cps*-type	MLST	Efficiency of plating			Dep-activity	
			KpS8	vB_KpnM_Seu621	vB_KpnP_Dlv622	DepS8	Dep622
Kp-9068^*^	K23	ST11	1.0	1.0	1.0	+	+
KPi4275^**^	K23	ST11	1.0	1.0	0.006	+	+
KPB2304-15	K23	ST11	1.0	1.0	0.006	+	+
KPB536-17-2	K23	ST1869	0.03	0.01	0.01	±	±
KPi1748	K2	ST65	−	−	−	−	−
KPi3014	K2	ST2174	−	−	−	−	−
KPi8289	K57	ST218	−	−	−	−	−
KPB2580	K1	ST23	−	−	−	−	−

* Host strain of vB_KpnP_Dlv622 and vB_KpnM_Seu621 phages.

** Host strain of KpS8 phage.

### Activity of Phage Depolymerases

To confirm whether the predicted proteins have polysaccharide-degrading activity, we cloned genes kps8_053 (phage KpS8) and dlv622_00059 (phage vB_KpnP_Dlv622) into the pET22b+ expression vector. His-tag fusion proteins DepS8 and Dep622 were expressed and purified.

**Figure 2:**
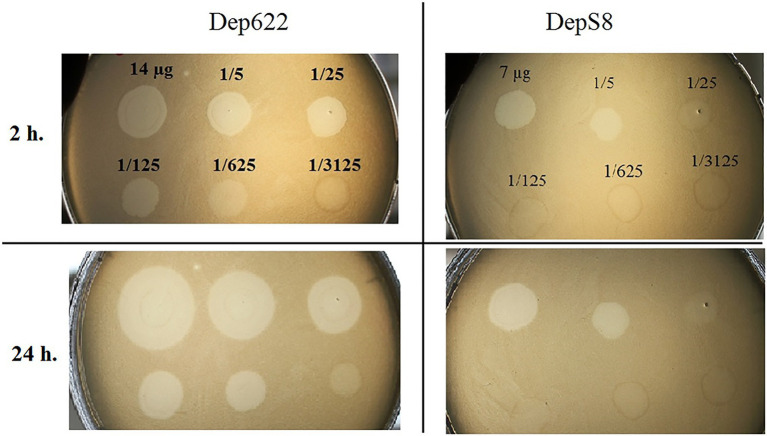
Spot tests of purified depolymerases DepS8 and Dep622 on *Klebsiella pneumoniae* Kp-9068 lawn.

The activities of DepS8 and Dep622 were tested on four isolates showing sensitivity according to the spot test and four control non-sensitive isolates. Both DepS8 and Dep622 recombinant depolymerases were able to form translucent spots resembling the plaque halo on four K23 isolates and showed a lack of activity for other isolates under investigation (Table 2; Figure 2).

### Depolymerases DepS8 and Dep622 Inhibit the Adsorption of Corresponding Bacteriophages

**Figure 3:**
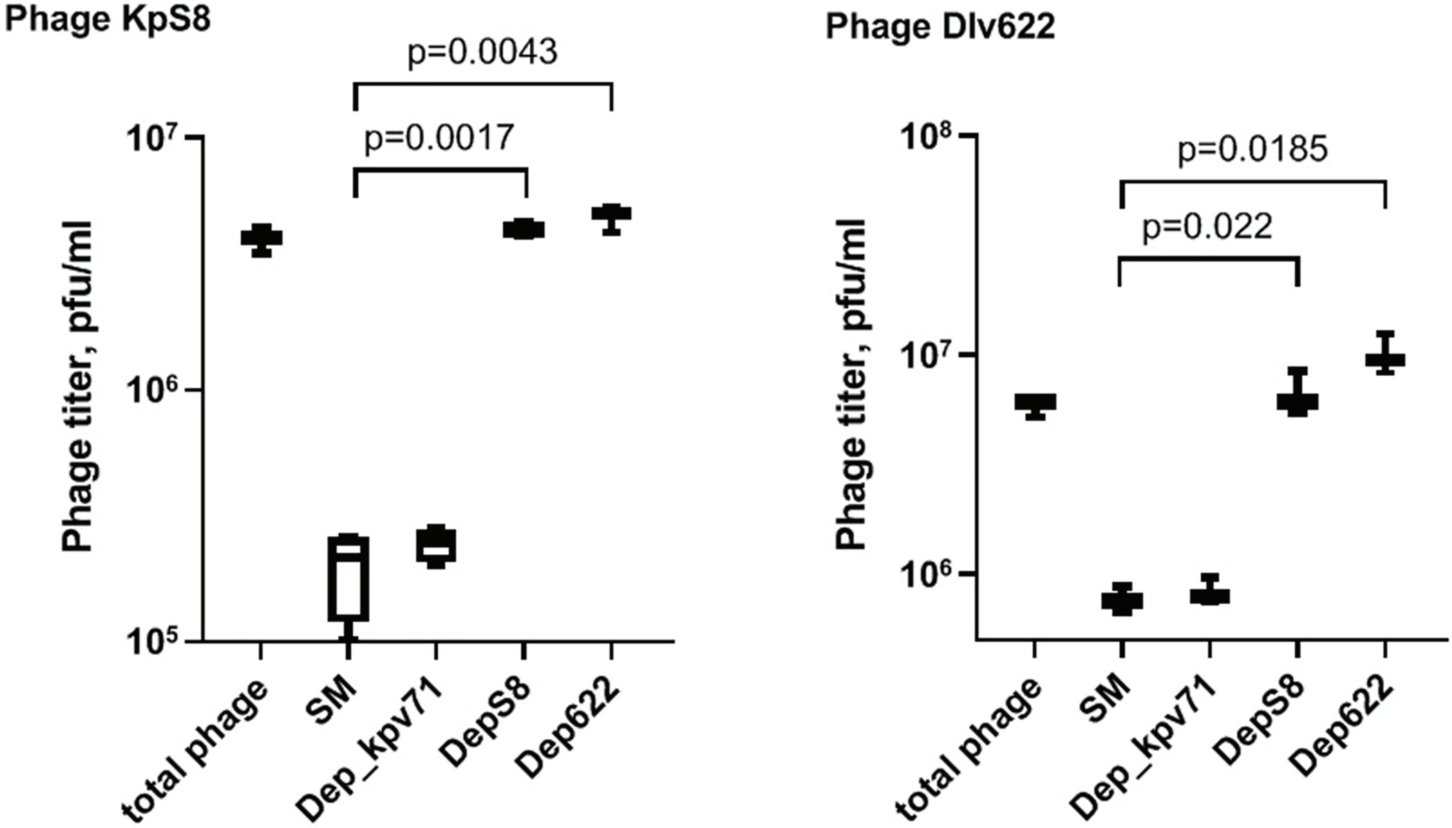
Phage KpS8 and vB_KpnP_Dlv622 adsorption inhibition assay. From left to right, a titer of the total phage sample and titers of the free phage particles after the incubation with *K. pneumoniae* Kp-9068 cells in SM and in the presence of Dep_kpv71 (K1 specific depolymerase), DepS8, and Dep622 are shown.

To study the interaction of bacterial viruses with surface receptors of a bacterial cell and determine whether the CPS depolymerases are the only key components to infect host cells, an adsorption inhibition assay was carried out. We used a competition assay to assess the role of DepS8 and Dep622 in the KpS8 and vB_KpnP_Dlv622 phage-cell interaction at the initial stages of the infection process. The K1 specific depolymerase Dep_kpv71 (Solovieva et al., 2018) was used as a control (Figure 3).

The assay showed a 10-fold decrease in phage titer in the control experiments with SM-buffer and with treatment by K1-specific recombinant depolymerase. On the contrary, treating bacterial cells with K23-specific depolymerases DepS8 and Dep622 did not lead to decrease in the titer of free phage. Both DepS8 and Dep622 inhibit the phage adsorption to *K. pneumoniae* cells, indicating that the phage and depolymerases compete for the same moieties on the host cell surface, which are capsular polysaccharides. These results are consistent with that DepS8 and Dep622 proteins were *in silico* predicted as putative tail fibers/spikes, responsible for the host cells’ recognition and reversible binding and subsequent degradation of *K. pneumoniae* cell capsular polysaccharides.

### Depolymerase Application Increases the Survival Rate of *K. pneumoniae* Kp-9068 Infected *G. mellonella* Larvae

The influence of DepS8 and Dep622 depolymerases on *K. pneumoniae* Kp-9068 virulence was estimated on the *G. mellonella* model by two parameters: 50% lethal dose of bacterial cells and survival curves following injection of *G. mellonella* larvae with fixed doses of *K. pneumoniae* Kp-9068 (3 × 10^5^ and 3 × 10^6^ CFU per injection).

**Figure 4:**
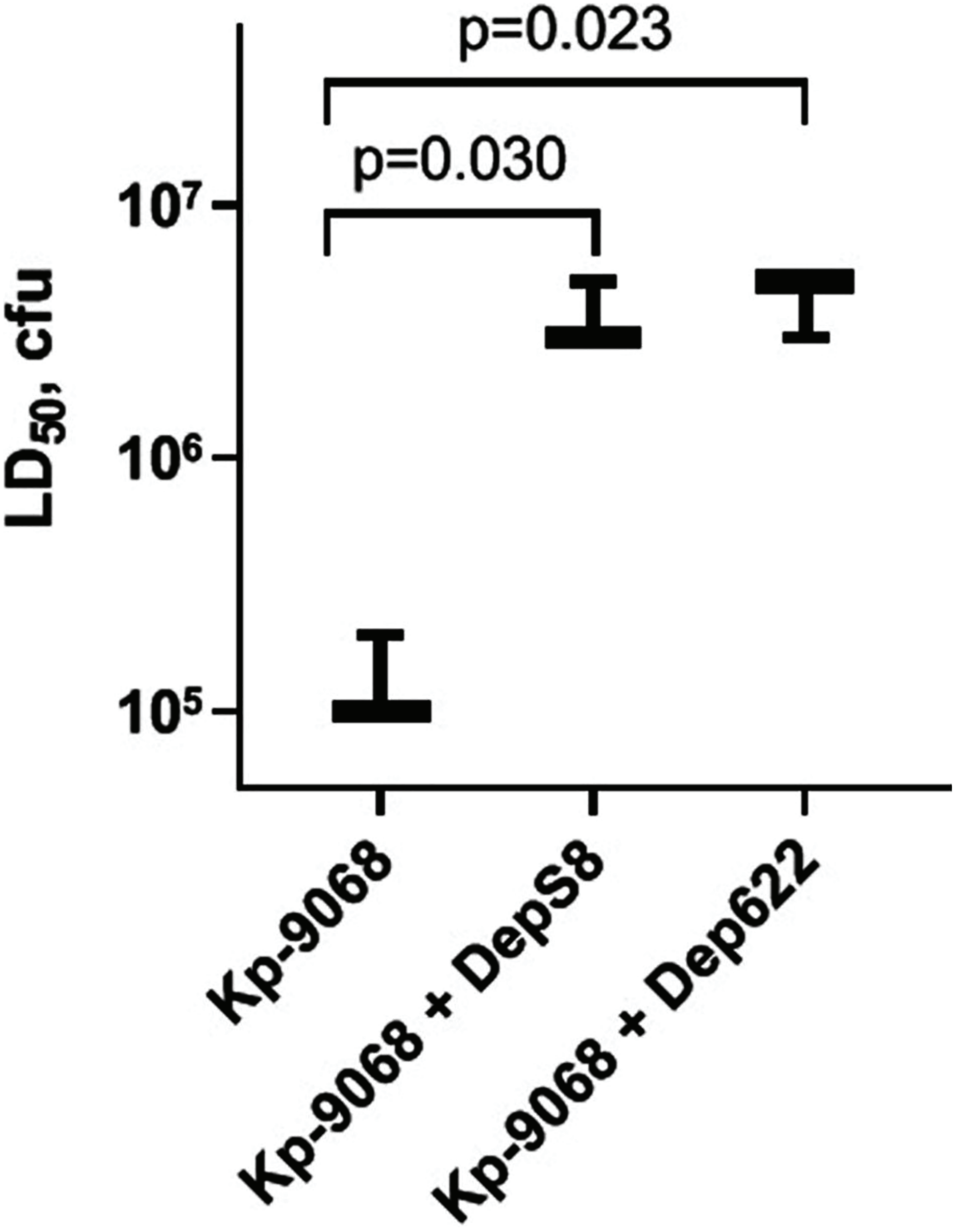
A dose of bacteria *K. pneumoniae* Kp-9068 is sufficient to kill 50% of the *Galleria mellonella* larvae population (LD_50_). From left to right, LD50 of the Kp-9068 untreated with depolymerase enzymes and LD_50_ of the Kp-9068 cells injected simultaneously with depolymerase DepS8 or Dep622. Average LD_50_ values from three independent experiments are shown and compared using Student’s *t*-test analysis. Values of *p* < 0.05 were considered as statistically significant.

Experiments have shown a decrease in the virulence of *K. pneumoniae* Kp-9068 cells that were treated with depolymerases. The median lethal dose (LD_50_) of untreated strain Kp-9068 was estimated as 1.3 × 10^5^ ± 5.8 × 10^4^ CFU. At the same time, LD_50_ for bacteria Kp-9068 treated with depolymerase DepS8 or Dep622 was higher by 27.5 and 32.5 times, respectively (Figure 4).

**Figure 5:**
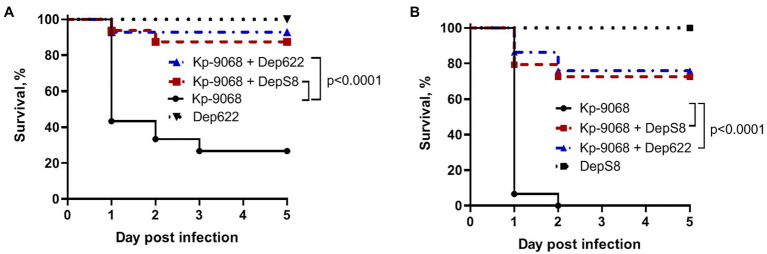
Kaplan–Meier survival curves following injection of *G. mellonella* larvae with *K. pneumoniae* Kp-9068 and Kp-9068 simultaneously with depolymerase enzymes. **(A)** Larvae treatment with 3 × 10^5^ CFU of *K. pneumoniae* Kp-9068; **(B)** Larvae treatment with 3 × 10^6^ CFU of *K. pneumoniae* Kp-9068. Larvae (*n* = 30) were injected with either bacteria and bacteria simultaneously with enzyme DepS8 or Dep622 (2 μg per larva). The experiments were controlled by observation of phosphate-buffered saline (PBS)-injected larvae and larvae receiving the depolymerase only. Survival for each control group was 100%, so for simplicity, a group of PBS-injected larvae was not included in the figure. Statistically significant differences in survival between larvae infected with bacteria only and larvae infected with bacteria simultaneously with depolymerase DepS8 or Dep622 were estimated by the log-rank (Mantel-Cox) test.

Without the treatment, 100% of the larvae died within 48 h after inoculation of 3 × 10^6^ CFU of *K. pneumoniae* Kp-9068 and 70% of the larvae died within 5 days after inoculation of 3 × 10^5^ CFU (Figure 5). In contrast, a single dose of each enzyme injected together with bacteria significantly inhibited *K. pneumoniae*-induced death in a time-dependent manner. Only 10–30% mortality was recorded within 5 days after inoculation of *K. pneumoniae* Kp-9068 together with depolymerase DepS8 or Dep622 (Figure 5). No mortality of larvae was observed in the controls, upon injection of PBS buffer, DepS8, or Dep622 enzymes alone.

## Discussion

Multidrug-resistant *K. pneumoniae* strains belonging to ST11 were used as host strains for the isolation of bacteriophages. Such isolates are widespread and are often associated with nosocomial outbreaks caused by carbapenem-resistant and hypervirulent strains in China (Gu et al., 2018) and Greece (Voulgari et al., 2014). Furthermore, the K23 capsular type is one of the 10 most common capsular types typically associated with nosocomial *K. pneumoniae* infection provoked by carbapenem-resistant strains (Follador et al., 2016). Isolates with capsular type K23 are detected on average in 4% of cases; however, among *K. pneumoniae* associated with the production of carbapenemase, the percentage of such isolates can reach 9–17% (Satlin et al., 2017; Sonda et al., 2018).

Within the context of research for a possible antimicrobial therapeutic agent, three novel lytic K23-specific *K. pneumoniae* phages were isolated. Phages KpS8 and vB_KpnM_Seu621 belonged to the *Myoviridae* family and had significant genetic similarities despite different times and sources of isolation (in 2016 from wastewater and in 2018 from river water). In turn, the vB_KpnP_Dlv622 phage belonged to the *Autographiviridae* family. Despite the considerable phylogenetic distance between the families, the bacteriophages possessed similar depolymerases and their corresponding recombinant proteins Dep622 and DepS8 exhibited the same rate of specificity and activity.

To search for similar cases, we analyzed proteins containing putative depolymerase domains for 76 *Klebsiella* phages, presented in NCBI (Volozhantsev et al., 2016, 2020; Labudda et al., 2017; Solovieva et al., 2018; Latka et al., 2019; Pan et al., 2019; Teng et al., 2019; Thiry et al., 2019; Wu et al., 2019; Domingo-Calap et al., 2020a,b; Gao et al., 2020; Horváth et al., 2020; Li et al., 2020; Zhang et al., 2020; Supplementary Figure 6). The depolymerase domains of our bacteriophages formed a separate cluster on the tree together with the BIS47_29 protein’s domain (GenBank: YP_009832536.1). Given the fact that according to BLASTp the BIS47_29 protein was similar to kps8_orf053 (100% coverage and 92.09% identity) and dlv622_orf00059 (98% coverage and 65.9% identity), we can assume that BIS47_29 depolymerase also has specificity against capsule type K23.

Confirmed cases in the detection of homologous depolymerases in phages belonging to different families are quite rare (Volozhantsev et al., 2020). Apart from depolymerases specific against the K57 capsule, described in our previous study (Volozhantsev et al., 2020), and depolymerases described in the current article, no similar cases of depolymerases encoded by phages from different families have been reported. In addition to the cases mentioned above, we can assume based on the phylogenetic analysis that similar patterns are observed for at least four other types of depolymerases specific against capsule types K1, K30/K69, K63, and KN2 (Supplementary Figure 6). Although some authors suggest the theoretical possibility of lateral transfer of RBPs or depolymerase domains (Latka et al., 2019), for the time being, there are no reliable ways to distinguish between lateral transfer and convergent evolution.

Due to the dose-dependent effect and the standardizability of application protocols, therapy with phage derivatives is actively discussed on a par with the therapy with virulent bacteriophages (Górski et al., 2020; Volozhantsev et al., 2020). Recombinant tail spike proteins of phages vB_KpnP_Dlv622 and KpS8 significantly reduced the mortality of *G. mellonella* larvae infected with the host strain supporting infection by these phages (Figure 5). As can be seen from the spot-test data, depolymerases are not less effective than phages directly, and they behave as a potent tool in the treatment of antibiotic-resistant *K. pneumoniae* infection. Depolymerases cleave the polysaccharides of bacterial cells but, unlike phages, do not possess a lytic activity and do not kill host bacteria. Their therapeutic effect is due to the fact that by cleaving the capsular polysaccharides of *K. pneumoniae*, depolymerase eliminates the defense of the bacterium against the immune system, thus creating conditions for complement-mediated killing (Lin et al., 2014; Majkowska-Skrobek et al., 2018; Liu et al., 2020; Volozhantsev et al., 2020), and phagocytosis by macrophages (Majkowska-Skrobek et al., 2018).

## Conclusion

Three new bacteriophages specific against *K. pneumoniae* with capsule type K23 were functionally characterized. Their depolymerases had significant similarity, despite the fact that bacteriophages belong to different families. Moreover, apart from the depolymerases specific against the K23 capsule, similar patterns were detected among K1-, K30/K69-, K57-, K63-, and KN2-specific depolymerases. Depolymerases derived from phages KpS8 and vB_KpnP_Dlv622 were equally efficient in cleaving capsular polysaccharides and also significantly reduced the mortality of *G. mellonella* larvae.

## Data Availability Statement

The datasets presented in this study can be found in online repositories. The names of the repository/repositories and accession number(s) can be found below: GenBank, MT939253, MT939252, and MT178275; BioProject, PRJNA705078.

## Author Contributions

RG, NV, and ES drafted the main manuscript and performed the data analysis. RG, NV, VK, IB, MK, NK, AP, GM, PS, VVV, ED, EK, MM, ID, EI, and ES planned and performed the experiments. RG, NV, AM, VAV, DB, and ES were responsible for bioinformatics analysis of data. All authors contributed to the article and approved the submitted version.

## Conflict of Interest

The authors declare that the research was conducted in the absence of any commercial or financial relationships that could be construed as a potential conflict of interest.

## Publisher’s Note

All claims expressed in this article are solely those of the authors and do not necessarily represent those of their affiliated organizations, or those of the publisher, the editors and the reviewers. Any product that may be evaluated in this article, or claim that may be made by its manufacturer, is not guaranteed or endorsed by the publisher.
