# Supplementary material for: Novel Klebsiella pneumoniae K23-Specific Bacteriophages From Different Families: Similarity of Depolymerases and Their Therapeutic Potential
Source: Front Microbiol. 2021 Aug 9;12:669618. doi: 10.3389/fmicb.2021.669618 (PMC8381472; doi:10.3389/fmicb.2021.669618)
Supplement: Supplementary file 1 [file Data_Sheet_1.ZIP › Supplementary Figures.docx]

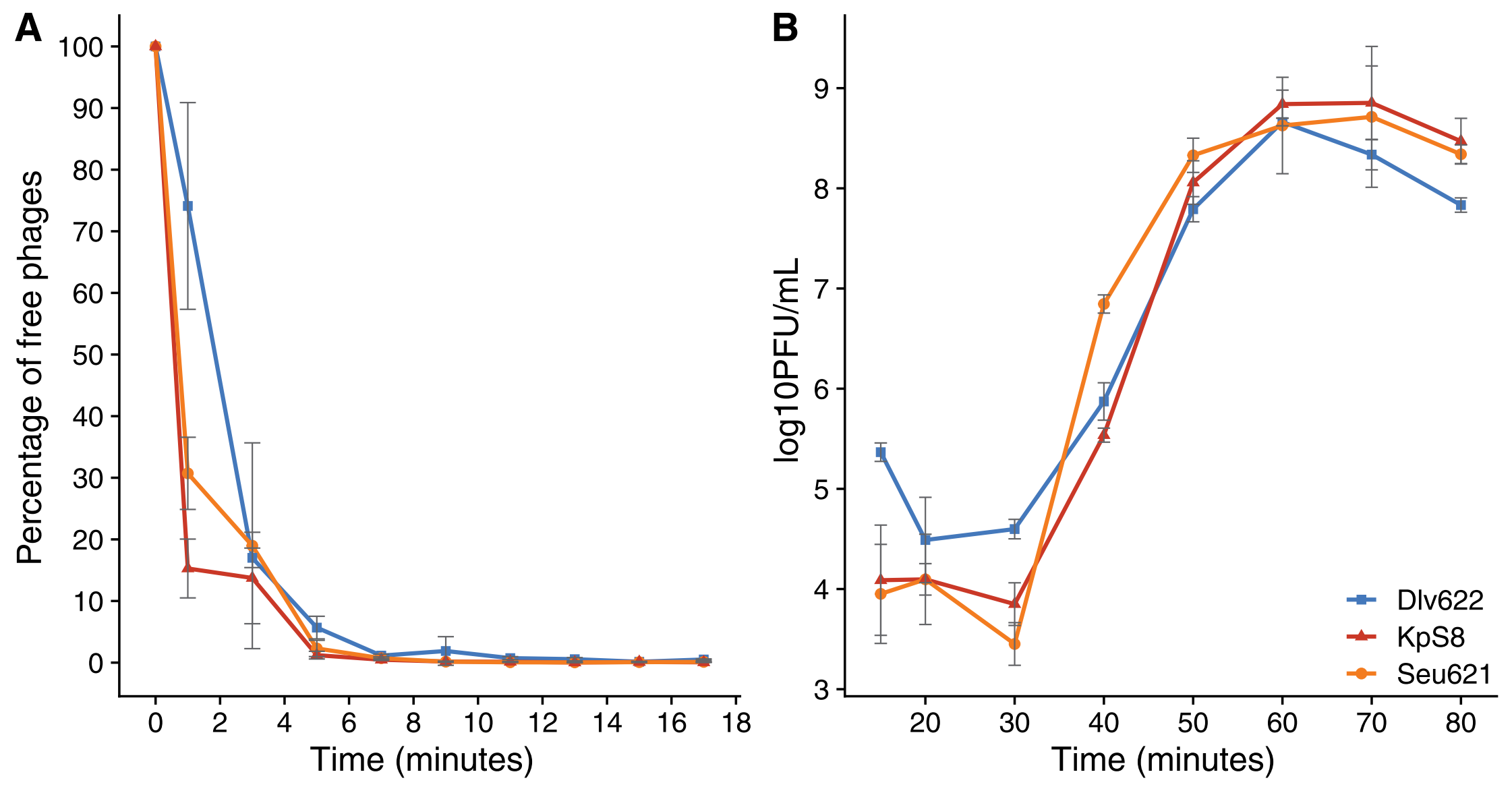
 **Supplementary Figure 1**. (A) Adsorption curve of bacteriophages vB_KpnЗ_Dlv622, vB_KpnM_Seu621, and KpS8. (B) One-step growth curve of bacteriophages vB_KpnЗ_Dlv622, vB_KpnM_Seu621, and KpS8.


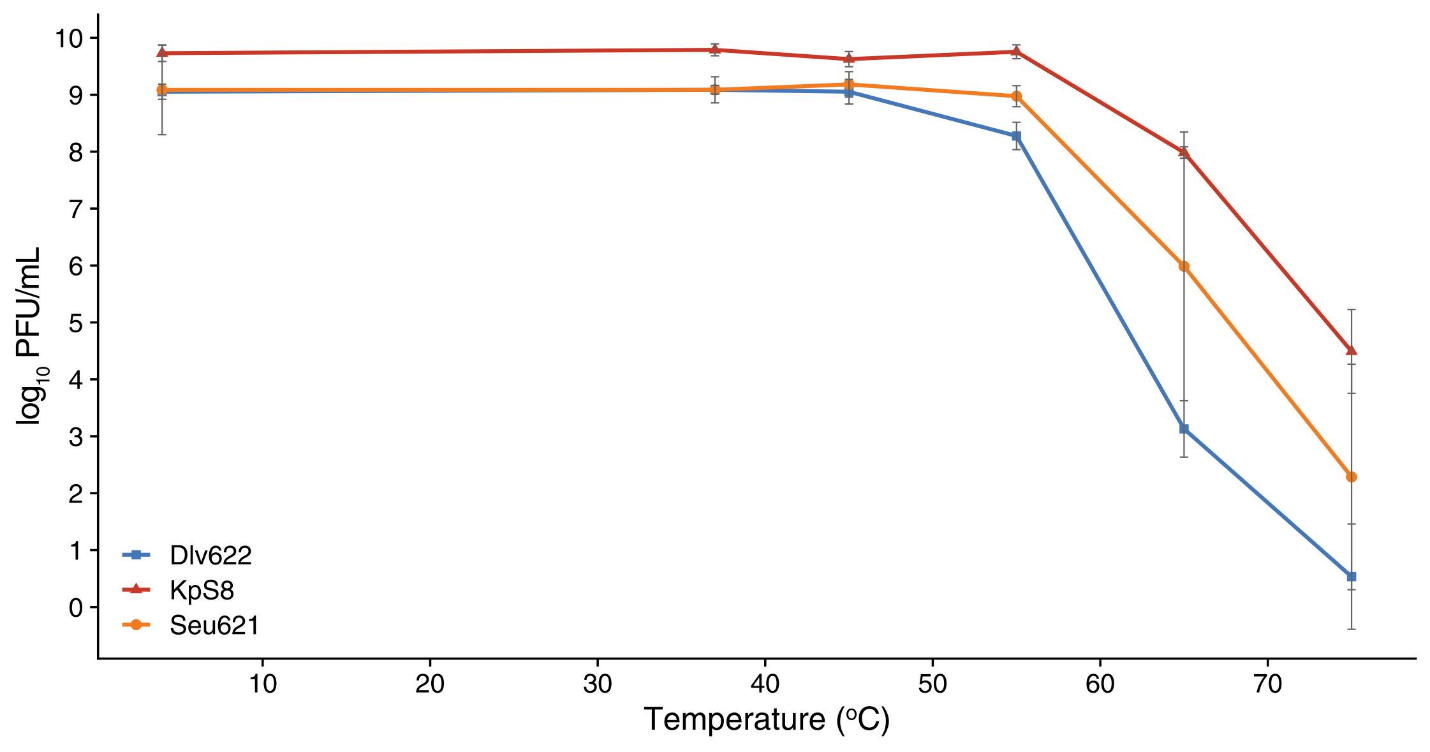


**Supplementary Figure 2**. Thermal tolerance of bacteriophages vB_KpnЗ_Dlv622, vB_KpnM_Seu621, and KpS8.


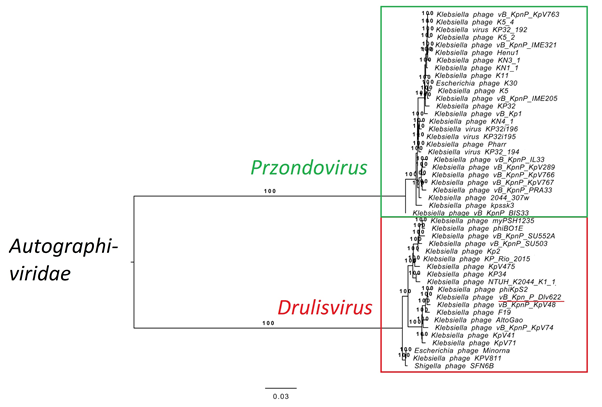


**Supplementary Figure 3**. Phylogenetic tree of *K. pneumoniae* *Autographiviridae* phages based on amino acid sequences of RNA polymerase.


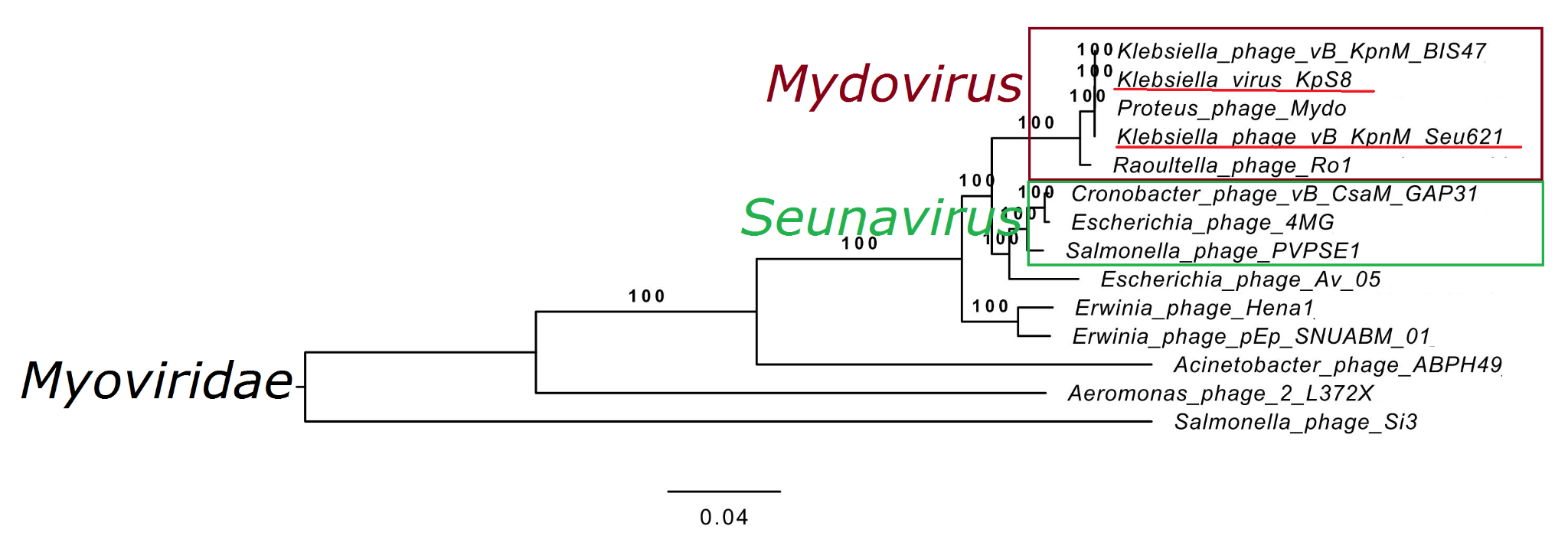


**Supplementary Figure 4**. Phylogenetic tree of *Myoviridae* phages based on amino acid sequences of major capsid protein.


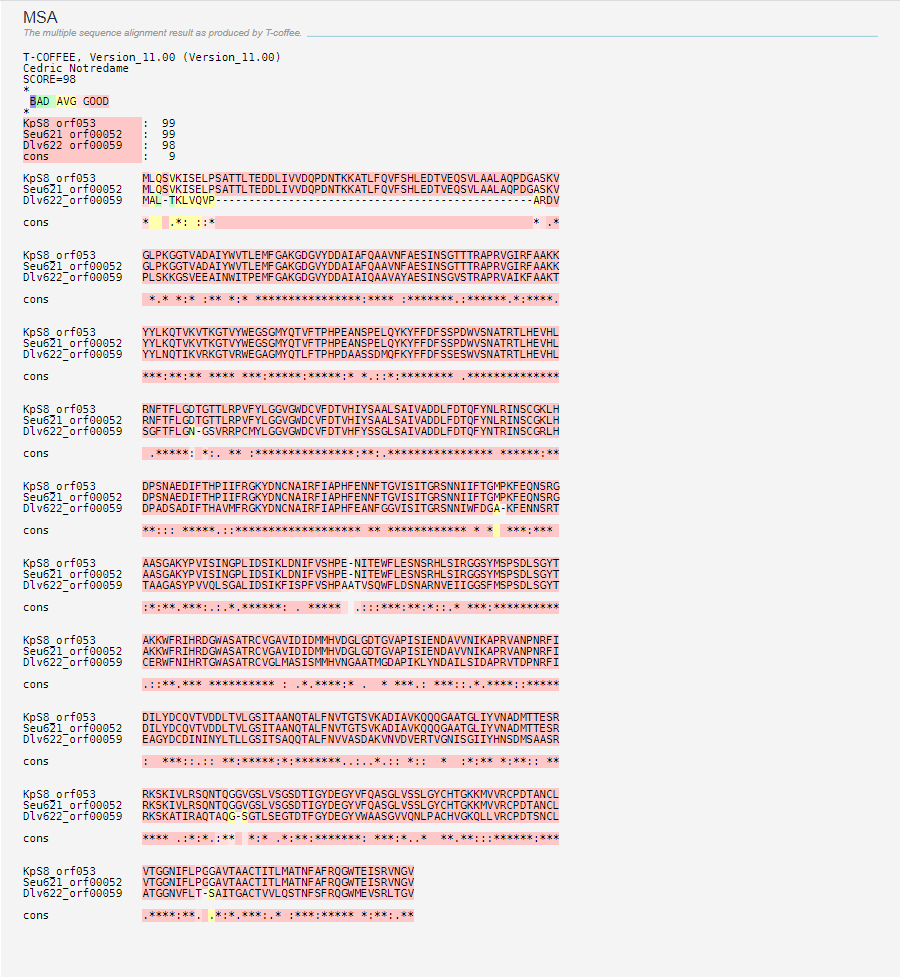


**Supplementary Figure 5.** Comparison* of the amino acid sequences of RBPs of vB_KpnP_Dlv622, vB_KpnM_Seu621, and KpS8 phages.

*Amino acid sequence alignment was done by the tcoffee algorithm (http://tcoffee.crg.cat/apps/tcoffee/index.html)


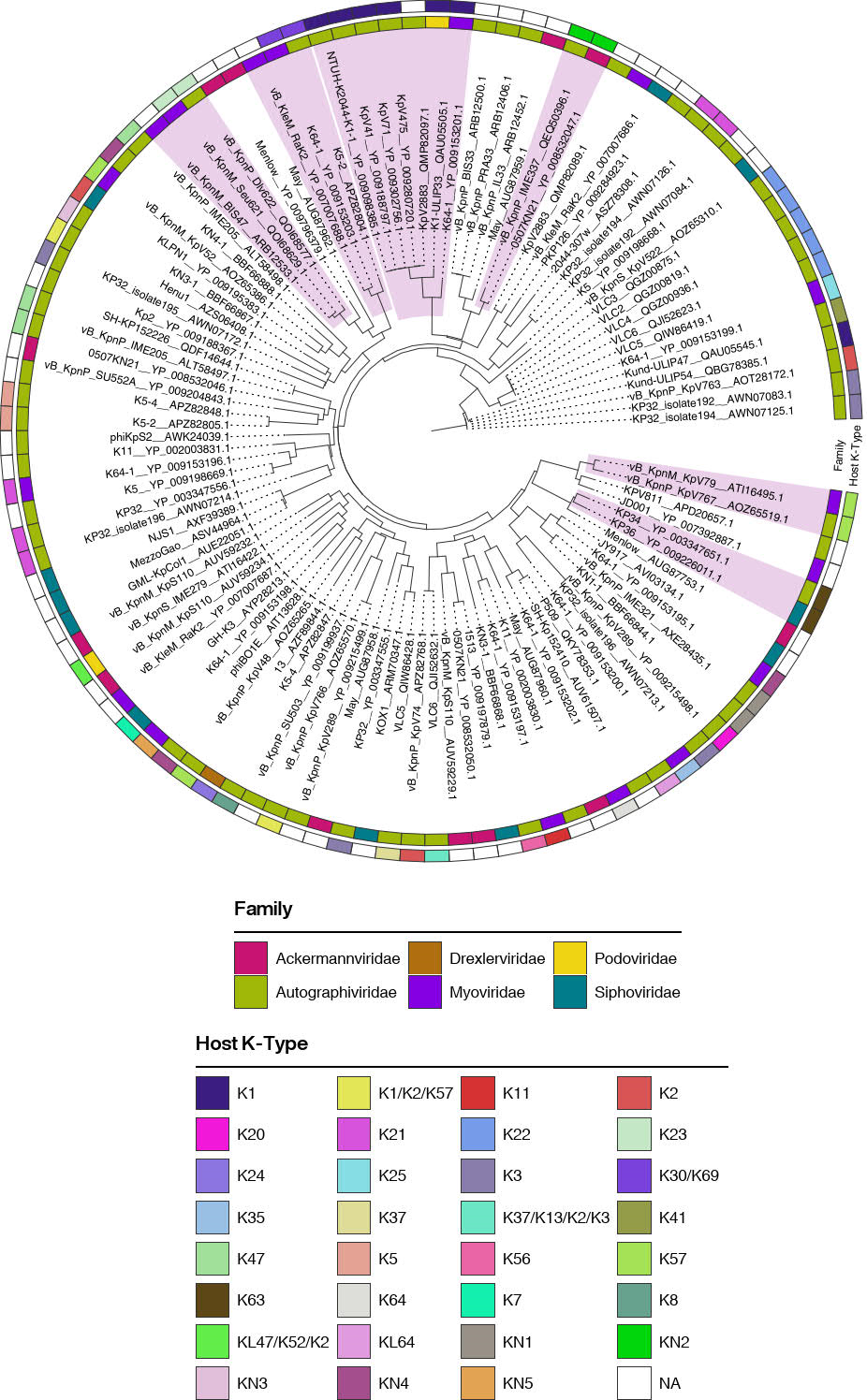


**Supplementary Figure 6**. Phylogenetic analysis of depolymerase domains of phage-encoded tail fiber proteins. Violet - depolymerases encoded by phages of different families and specific against the same capsular type.
